# Neoadjuvant Chemotherapy in Triple Negative Breast Cancer: A Systematic Review of Breast and Node Pathologic Response

**DOI:** 10.1002/jso.70213

**Published:** 2026-02-15

**Authors:** Milena Martello Cristófalo, Jonathan Yugo Maesaka, Deise Azevedo Pereira, Gabriela Bezerra Nóbrega, Yedda Nunes Reis, José Maria Soares Júnior, Edmund Chada Baracat, José Roberto Filassi

**Affiliations:** ^1^ Institute of Cancer of the State of São Paulo, School of Medicine University of São Paulo São Paulo Brazil; ^2^ Memorial Arthur Ramos Hospital—rede D'Or, R. Hugo Corrêa Paes Maceió Brazil

**Keywords:** breast cancer, neoadjuvant chemotherapy, pathological response, systematic review, triple negative

## Abstract

**Introduction:**

Pathological complete response (pCR) after neoadjuvant chemotherapy is associated with improved prognosis in patients with triple‐negative breast cancer (TNBC). Differences in pathological response rates between the breast and axillary lymph nodes have prompted interest in understanding response patterns that may, in the future, inform strategies aimed at omitting axillary surgical evaluation. This systematic review aimed to describe and compare the prevalence of breast and axillary pathological responses in TNBC patients treated with neoadjuvant chemotherapy.

**Methods:**

This systematic review was conducted following the PRISMA statement and registered in PROSPERO (ID: CRD498121). Searches were performed in the PubMed, Embase, and Web of Science databases. Studies that described node pathological response (NpCR) and breast pathological response (BpCR) in TNBC patients undergoing neoadjuvant chemotherapy were included. Article selection was independently performed by two reviewers using the Rayyan platform. The methodological quality of the included studies was assessed using the Newcastle‐Ottawa Scale.

**Results:**

Across the included studies, NpCR rates were consistently higher than BpCR rates in TNBC patients. No study reported higher BpCR compared with NpCR. The mean prevalence of BpCR was 32% (SD 0.6), NpCR was 38.3% (SD 0.9).

**Conclusion:**

Among TNBC patients treated with neoadjuvant chemotherapy, NpCR occurs more frequently than BpCR. These findings provide a descriptive overview of current response patterns and may inform future research exploring the safety of omitting axillary surgical evaluation. Factors beyond tumor subtype likely influence response patterns, indicating the need for further research to identify predictive biomarkers and optimize treatment strategies.

## Summary

- Axillary pathological response (NpCR) is higher than breast response in triple‐negative breast cancer. Mean pathological breast response: 32% (SD 0.6); mean node response: 38.3% (SD 0.9).
- Pathological breast response (BpCR) does not ensure a complete response in triple‐negative breast cancer.
- Factors beyond the tumor subtype may affect the pattern of pathological response to chemotherapy.

## 1 Introduction

Pathological response to neoadjuvant chemotherapy is strongly associated with disease prognosis [1]. Pathological complete response (pCR) is defined as the absence of residual invasive disease in the breast and axillary lymph nodes, with or without residual in situ carcinoma following neoadjuvant treatment (ypT0/ypTis/ypN0). In the axilla, pCR also requires the absence of isolated tumor cells and micrometastases [2].

Achieving axillary pCR (NpCR) is associated with improved disease‐free and overall survival and has supported efforts toward less invasive axillary management [3, 4]. Landmark trials such as SENTINA [5], ACOSOG Z1071 [6], and FNAC [7] have validated the use of sentinel lymph node biopsy (SLNB) after neoadjuvant chemotherapy in selected patients with clinically negative axilla, allowing omission of axillary lymph node dissection and reducing morbidity, including lymphedema, sensory deficits, and chronic pain [8].

Among breast cancer subtypes, triple‐negative breast cancer (TNBC) represents a biologically aggressive and heterogeneous disease, characterized by the absence of estrogen receptor, progesterone receptor, and HER2 expression [9]. Despite its aggressive clinical behavior, TNBC demonstrates high sensitivity to systemic therapy, with some studies reporting overall pCR rates of up to 65% following neoadjuvant treatment [10]. This marked chemosensitivity has positioned TNBC as a key model for investigating response‐adapted treatment strategies, including the future possibility of surgical omission [11, 12].

Several clinical and biological factors have been associated with achieving pCR, including initial axillary status, immunohistochemical subtype with hormone receptor negativity, and breast response following neoadjuvant chemotherapy [13]. In this context, increasing interest has emerged in understanding whether breast pCR (BpCR) may correlate with axillary response (NpCR) in TNBC, as such an association could, in the future, inform more conservative axillary surgical approaches.

Therefore, this systematic review aims to evaluate and summarize the available scientific literature on the prevalence of NpCR and BpCR in TNBC patients treated with neoadjuvant chemotherapy.

## 2 Methods

This systematic review was registered in PROSPERO (ID: CRD 420250498121) [14] and conducted in accordance with the PRISMA guidelines [15].

### 2.1 Eligibility Criteria

Observational studies evaluating BpCR and NpCR following neoadjuvant treatment in patients with TNBC were included (Table 1). No restrictions were applied regarding the type of chemotherapy regimen used or the type of surgery performed.

**Table 1:** PECOS Guideline.

PECOS guideline	Definition
Population	Women with a histopathological diagnosis of triple‐negative breast cancer
Exposure	Neoadjuvant chemotherapy
Comparison	—
Outcome	Pathological response in the breast and axilla
Study design	Observational studies (cohort or cross‐sectional)

### 2.2 Information Sources

The PubMed, EMBASE, and WEB OF SCIENCE databases were consulted. The selected articles were published in English, Spanish, and Portuguese.

### 2.3 Search Strategy and Selection Process

The search strategies used in this review, including articles published up to November 2023, are available in Supporting Information S1: 1. Studies were selected based on title and abstract analysis to assess the relevance of the full text. The Rayyan application from the Qatar Computing Research Institute [16] was used to facilitate the evaluation process, ensuring reviewer blinding through the “blind on” option available in the tool. All articles were independently reviewed in full by two evaluators (D.P.A. and G.B.N.), both breast surgeons. Discrepancies in study selection were resolved through discussion with a third evaluator (J.Y.M.).

### 2.4 Data Collection and Data Items

Data from the selected articles were extracted and stored in a customized table containing the following variables: total number of patients, age, menopausal status, histopathological and immunohistochemical tumor type, surgical procedure performed, prescribed chemotherapy regimen, initial staging, method of axillary involvement assessment, and prevalence of clinical and complete pathological response in the breast and axilla.

### 2.5 Methodological Quality Assessment

The methodological quality of the included studies was independently assessed by two reviewers using the Newcastle‐Ottawa Scale (NOS) for cohort studies [17]. NOS evaluates aspects such as sample selection, comparability of study groups, and outcomes. Studies with a score of 6 or higher were considered to have adequate quality.

### 2.6 Effect Measures and Synthesis Methods

Data extracted from individual studies were analyzed both qualitatively and quantitatively. The pCR was quantified as the mean and standard deviation.

## 3 Results

### 3.1 Study Selection

**Figure 1:**
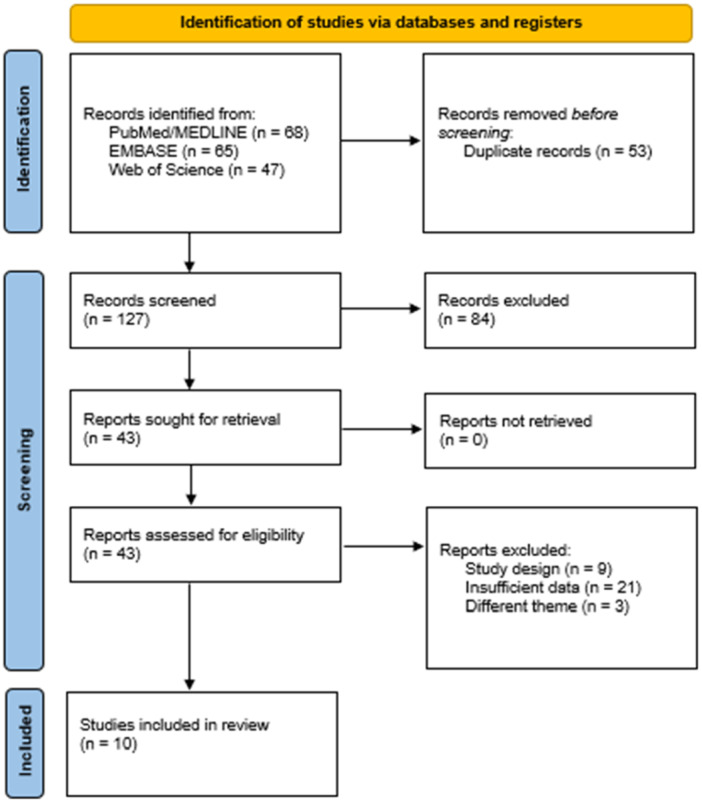
PRISMA flowchart for article selection in the systematic review.

Until November 2023, 180 articles were retrieved from the three databases consulted. Among them, 53 were excluded due to duplication after export to the systematic review organization tool, and 84 did not meet the pre‐defined selection criteria. Thus, 43 articles were selected for full‐text reading. After completing the reading of these 43 articles, 33 were excluded for not meeting the selection criteria, and 10 were considered eligible to be included in the present systematic review (Figure 1).

### 3.2 Study Characteristics

The retrieved articles, consisting of 10 cohort studies (Table 2), included an evaluation of 24 332 patients diagnosed with malignant breast neoplasia, with invasive carcinoma of no special type being the predominant histological subtype in postmenopausal patients with stage II disease.

**Table 2:** General characteristics of the included studies.

	Zhang et al. [26]	Boughey et al. [22]	Tadros et al. [18]	Fayanju et al. [23]	Resende et al. [19]	Cerbelli et al. [20]	Kim et al. [21]	Karalink 2021	Myers et al. [24]	Jankowski et al. [27]
Patients	301	694	527	20265	310	181	244	25	1348	437
Age (median)	46 (19−75)	—	51 (23−84)	52 (44−60)	—	—	—	46 (26−74)	54 (44−63)	52 (39−64)
Post‐menopause	—	345 (49.7%)	298 (56.5%)	—	162 (52%)	102 (56.4%)	118 (48,4%)	8 (32%)	736 (55%)	213 (48.7%)
Tumor histologic type	Ductal (94%)	—	Ductal (98%)	Ductal (85%)	—	Ductal (94%)	Ductal (94%)	Ductal (76%)	Ductal (80%)	Ductal (95%)
Chemotherapy	Anthracycline and/or taxane and/or platinum	Anthracycline and/or taxane	Anthracycline and taxane	—	Anthracycline and taxane	Anthracycline and taxane	Anthracycline and/or taxane	Anthracycline and taxane	—	Anthracycline and/or taxane
TNBC	55 (18%)	170 (24%)	264 (50%)	6246 (31%)	94 (30%)	31 (17%)	47 (19%)	4 (16%)	197 (15%)	98 (22%)
cN + TNBC before chemo	26/55 (47%)	170/170 (100%)	106/264 (40%)	2368/6246 (40%)	65/94 (69%)	31/31 (100%)	47/47 (100%)	4/4 (100%)	79/197 (40%)	98/98 (100%)
Axillary evaluation	Physical exam	FNA/CORE	FNA/CORE	Physical exam	CORE	FNA/CORE	CORE	Physical exam/FNA	Physical exam	Physical exam/FNA/CORE
Breast surgery	253 (84%) Mastectomy	415 (60%) Mastectomy	276 (52%) Mastectomy	12557 (62%) Mastectomy	—	253 (84%) Mastectomy	152 (62%) Mastectomy	25 (100%) Mastectomy	713 (53%) Mastectomy	244 (56%) Mastectomy
Axillary surgery	Axillary dissection	SLNB + Axillary dissection	SLNB or Axillary dissection	SLNB or Axillary dissection	—	SLNB or Axillary dissection	Axillary dissection	SLNB + Axillary dissection	SLNB or Axillary dissection	Axillary dissection
Stage	II	II	III	II	III	II	II	II	II	II
NOS	7	8	7	8	8	7	7	6	7	8

Four different immunohistochemical subtypes were classified, with each patient assigned to only one subtype: hormone receptor‐positive/HER2‐negative (HR+/HER2‐ or luminal), hormone receptor‐positive/HER2‐positive (HR+/HER2+), hormone receptor‐negative/HER2‐positive (HER2+), and hormone receptor‐negative/HER2‐negative (triple‐negative).

Pre‐treatment axillary involvement was documented in 11 490 patients. In five studies (1584 patients) [18, 19, 20, 21, 22], axillary involvement was confirmed through cytological evaluation before the initiation of systemic treatment. In the remaining studies (9906 patients) [23, 24, 25, 26, 27], axillary positivity was determined based on findings from physical examination, imaging results, and/or cytology and/or histological assessment.

BpCR was defined as the absence of invasive disease (ypT0/ypTis) in six studies [19, 20, 21, 22, 26, 27]. In the remaining studies, it was defined as the absence of both invasive and in situ disease (ypT0) [18, 23, 24, 25]. NpCR was defined as the absence of invasive tumor cells in axillary lymph nodes (ypN0) in all evaluated studies.

Mastectomy was the most common breast surgery procedure performed across all studies. In five studies, axillary dissection was routinely performed in all patients with [22, 25] or without [21, 26, 27] prior SLNB. However, it is worth noting that the study by Karanlik et al. [25] included patients with inflammatory breast carcinoma, for which axillary lymph node dissection remains the standard surgical approach. In four studies, patients could undergo SLNB without subsequent axillary dissection [18, 20, 23, 24]. One study did not specify the type of breast or axillary surgery performed [19]. Notably, none of the included studies reported the proportion of patients with germline mutations, limiting the assessment of potential genetic influences on surgical decision‐making.

### 3.3 Methodological Quality Assessment

Each of the 10 previously selected articles was subjected to bias assessment according to the NOS scale. All 10 selected articles scored 6 or higher, representing acceptable quality in their methodology (Table 2).

### 3.4 Study Results

The 10 studies included 7206 patients (30%) with the immunohistochemical subtype TN, of whom 2994 (42%) had a positive axilla (cN+) prior to neoadjuvant chemotherapy. Among these, 419 had cN+ confirmed by cytological evaluation, while axillary positivity in the remaining cases was defined based on clinical assessment or imaging findings. In four studies [20, 21, 22, 27], all patients included had cN+, as the inclusion criteria of these authors only admitted patients with this characteristic. In the remaining studies [18, 19, 23, 24, 25, 26], axillary positivity ranged from 40% to 69%.

BpCR in the TN subtype occurred in 2291 patients, ranging from 19% to 48% across studies, with a mean of 32% (SD = 0.6). NpCRoccurred in 1148 patients, ranging from 25% to 92%, with a mean of 38.2% (SD = 0.9). Regarding overall pCR, the mean rate was 40% (SD = 0.6) (Table 3). However, it is important to consider that overall pCR was defined as ypT0/ypTis. That is, patients whose axilla was initially negative and remained negative were also classified as having overall pCR, which may explain the higher mean overall pCR compared with BpCR or NpCR rates.

**Table 3:** Triple negative breast cancer pathological response.

	Zhang et al. [26]	Boughey et al. [22]	Tadros et al. [18]	Fayanju et al. [23]	Resende et al. [19]	Cerbelli et al. [20]	Kim et al. [21]	Karalink [28]	Myers et al. [24]	Jankowski et al. [27]
BpCR	18/55 (33%)	81/170 (48%)	99/264 (38%)	1936/6246 (31%)	26/94 (28%)	11/31 (35%)	9/47 (19%)	1/4 (25%)	70/197 (36%)	40/98 (41%)
NpCR	24/26 (92%)	84/170 (49%)	54/106 (51%)	829/2368 (35%)	40/65 (62%)	13/31 (42%)	20/47 (43%)	1/4 (25%)	37/79 (47%)	46/98 (47%)
pCR	14/55 (25%)	65/170 (38%)	96/264 (36%)	2611/6246 (30%)	21/94 (22%)	9/31 (29%)	8/47 (17%)	1/4 (25%)	27/197 (14%)	32/98 (33%)

The percentage of NpCR was higher than BpCR in nine studies [18, 19, 20, 21, 22, 23, 24, 26, 27], and in only one study were these percentages equal [25]. In no study was BpCRhigher than NpCR.

When pathological response rates were compared across immunohistochemical subtypes, HER2‐positive tumors most frequently achieved the highest pCR rates, followed by triple‐negative tumors, whereas luminal tumors consistently showed the lowest response rates across all outcomes (Supporting Information S1: 2, 3, 4).

## 4 Discussion

This systematic review, including 7206 patients with TNBC, demonstrated a higher axillary response rate compared to the breast response rate in patients who underwent neoadjuvant chemotherapy.

pCR provides prognostic information of notable clinical relevance, as consistently demonstrated in previous studies. Patients with BpCR or NpCR have better overall survival than those without a response [14]. Conversely, they have worse survival compared to those with overall pCR [18, 23]. Therefore, pCR is an important prognostic biomarker when evaluating systemic treatment [14].

The molecular subtype is a predictor of pCR, incorporating relevant biological data such as estrogen receptor status, HER2 status, and Ki67 levels [20, 29]. In this context, the higher predictive power for pCR in TN tumors and their chemosensitivity can be explained by their biological characteristics, such as negative estrogen receptor status and higher Ki67 expression, which are also associated with high tumor aggressiveness—the so‐called “triple‐negative tumor paradox” [19]. Additionally, the higher likelihood of achieving pCR in TN tumors is also related to staging [18]. The smaller the tumor burden in the breast and axilla, the greater the chance of pCR. The use of targeted therapies in combination with chemotherapy also increases the chance of pCR, as in the case of TN tumors that achieved a 65% rate (ypT0/is ypN0) with the addition of pembrolizumab to conventional systemic therapy [10].

The data retrieved in this review indicate a BpCR rate of 32% (SD 0.6) and NpCR rate of 38.3% (SD 0.9) for TN tumors. Notably, previous reviews have also specifically evaluated NpCR rates in TN tumors and reported similar findings [4]. In general, the percentage of NpCR is significantly higher than that of BpCR, a characteristic related to the greater heterogeneity of tumor cells in the breast and lower chemosensitivity [27]. In TN tumors, although NpCR is generally higher than BpCR, it cannot be guaranteed that patients who achieve BpCRwill also achieve NpCR. In other words, BpCR cannot be interpreted as the sole predictor of NpCR. It is possible that other factors, yet to be studied, may assist in this assessment.

Beyond tumor burden and treatment‐related factors, the biological heterogeneity of TNBC may also contribute to the observed variability in pathological response patterns between the breast and axilla [30]. Genomic classifiers such as BluePrint have identified several subsets of TNBC, including basal‐like immune‐activated, basal‐like immunosuppressed, luminal androgen receptor, and mesenchymal [31], which may exhibit differential sensitivity to systemic therapies. These findings suggest that immune‐related factors, hormone receptor signaling pathways, and tumor microenvironment characteristics may play a role in modulating pathological response beyond conventional clinicopathologic variables. While the present review was not designed to stratify outcomes according to TNBC molecular subtypes, recognizing this heterogeneity is essential and underscores the need for future prospective studies integrating molecular classification, immune biomarkers, and treatment response to better inform personalized therapeutic strategies in TNBC.

Given the excellent pCR rates in TN tumors, the need for breast surgery has been questioned when post‐neoadjuvant treatment biopsy indicates the absence of invasive disease [11, 12]. However, the optimal management of the axilla in these situations remains unknown, pending the results of prospective clinical trials such as ASICS that are specifically designed to address this question [32]. None of the studies could suggest that BpCR can reliably predict the pattern of axillary response. On the other hand, they emphasized the possibility of less invasive management, particularly through the use of dual‐tracer SLNB with excision of at least three lymph nodes [5, 7, 22]. In parallel, alternative approaches such as targeted axillary dissection, combining SLNB with the removal of a previously marked positive node, have also been proposed to enhance staging accuracy while reducing surgical morbidity [33, 34].

The main limitations of this study include the lack of uniformity in the chemotherapy regimens used and the absence of immunotherapy in the included studies. None of the articles evaluated patients treated with immunotherapy, primarily due to the limited availability of these agents during the periods in which the studies were conducted. It is, however, well established that the addition of immunotherapy to neoadjuvant chemotherapy results in higher pCR rates in TNBC and is currently considered standard of care in the neoadjuvant setting, regardless of tumor‐infiltrating lymphocyte (TIL) status [10]. There was also no homogeneity in the diagnostic method for axillary involvement, whether by clinical, cytological, or histopathological examination, which may introduce bias into the presented data. Furthermore, it is important to highlight that not all patients underwent axillary lymph node biopsy prior to the initiation of chemotherapy, making it difficult to accurately assess the initial extent of nodal disease and potentially affecting response evaluation. Finally, it is worth noting the type of breast surgery performed in the reported studies. Although most of the evaluated patients were diagnosed at an early stage (stage II), radical surgery was the preferred procedure. However, patients with TN tumors benefit from systemic treatment, even at an early stage, tend to exhibit pathological response, and benefit from conservative surgery in the breast and axilla [35].

Despite its limitations, this systematic review presents several strengths. First, it reinforces the finding that axillary pathological response is more frequent than breast response in patients with triple‐negative tumors. Second, in the current context of surgical de‐escalation, systematic reviews focusing on pathological response patterns gain particular importance, as they offer critical insights into tumor behavior and treatment efficacy. Lastly, understanding the nuances of pathological response in both the breast and axilla is essential for designing and supporting future studies aimed at safely minimizing surgical interventions, especially in subtypes with high chemosensitivity, such as TNBC.

This systematic review raises an important question that may be answered in future clinical trials, better understanding of other factors that may be involved in the pattern of pathological response for triple‐negative tumors. At present, surgical axillary staging after neoadjuvant therapy remains essential in breast cancer treatment, providing prognostically relevant clinical information.

## 5 Conclusion

Despite high chemosensitivity in TNBC, rates of BpCR are consistently lower than the rates of NpCR. Factors beyond tumor subtype likely influence response patterns, indicating the need for further research to identify predictive biomarkers and optimize treatment strategies.

## Conflicts of Interest

The authors declare no conflicts of interest.

## Synopsis

This systematic review evaluated and compared pathological response rates in the breast and axillary lymph nodes of patients with triple‐negative breast cancer treated with neoadjuvant chemotherapy. Across studies, axillary pathological response was consistently more frequent than breast response, with no reports of higher breast response than nodal pathological response. These findings underscore distinct patterns of response to chemotherapy and highlight the need for further research to clarify their clinical implications and identify factors influencing breast and nodal treatment response.

## Supporting information

SUPPLEMENTARY.

## Data Availability

The authors have nothing to report.
