# Supplementary material for: Neoadjuvant Chemotherapy in Triple Negative Breast Cancer: A Systematic Review of Breast and Node Pathologic Response
Source: J Surg Oncol. 2026 Feb 15;133(6):712–9. doi: 10.1002/jso.70213 (PMC13128993; doi:10.1002/jso.70213)
Supplement: Supplementary file 1 — SUPPLEMENTARY. [file JSO-133-712-s001.docx]

**SUPPLEMENTARY**

Supplementary 1 - Search Strategy for Each Research Database

| Database | Search |
| --- | --- |
| MEDLINE / PUBMED | ((triple negative breast cancer) and (neoadjuvant chemotherapy)) and (lymph node)) and (axilla response)) and (breast response)) |
| EMBASE | ('triple negative breast cancer'/exp OR 'triple negative breast cancer') AND 'neoadjuvant chemotherapy' AND 'lymph node' AND axilla AND breast AND response) |
| WEB OF SCIENCE | triple negative breast cancer (todos os campos) and neoadjuvant chemotherapy (todos os campos) and lymph node (todos os campos) and axilla response (todos os campos) and breast response (todos os campos) |

Supplementary 2 - Breast pCR according to tumor subtypes (ypT0/ypTis)

|  | **Zhang 2013** | **Boughey 2014** | **Tadros 2017** | **Fayanju 2018** | **Resende 2018** | **Cerbelli 2019** | **Kim 2019** | **Karalink 2021** | **Myers 2021** | **Jankowski 2023** |
| --- | --- | --- | --- | --- | --- | --- | --- | --- | --- | --- |
| HR+ / HER2 - | 29/145  (20%) | 49/317  (15%) | --- | 866/8244  (10%) | 4/142  (3%) | Luminal A 1/18  (6%)  Luminal B 15/65  (23%) | 14/114  (12%) | 2/12  (17%) | 24/155  (15%) | 9/176  (5%) |
| HR+ / HER2+ | --- | --- | --- | 925/3628  (25%) | --- | 7/30  (23%) | 10/39  (26%) | 2/5  (40%) | 133/653  (20%) | 30/91  (33%) |
| HR- / HER2+ | 50/101  (49%) | 103/207  (50%) | 94/263  (36%) | 966/2147  (45%) | 21/74  (28%) | 22/37  (59%) | 15/44  (34%) | 4/4  (100%) | 147/343  (43%) | 39/72  (54%) |
| HR- / HER2- | 18/55  (33%) | 81/170  (48%) | 99/264  (38%) | 1936/6246 (31%) | 26/94  (28%) | 11/31  (35%) | 9/47  (19%) | 1/4  (25%) | 70/197  (36%) | 40/98  (41%) |

Supplementary 3 - Node pCR according to tumor subtypes (N+ → ypN0)

|  | **Zhang 2013** | **Boughey 2014** | **Tadros 2017** | **Fayanju 2018** | **Resende 2018** | **Cerbelli 2019** | **Kim 2019** | **Karalink 2021** | **Myers 2021** | **Jankowski 2023** |
| --- | --- | --- | --- | --- | --- | --- | --- | --- | --- | --- |
| **HR+ / HER2 -** | 50/84  (59%) | 67/317  (21%) | --- | 528/3522  (15%) | 23/106  (22%) | Luminal A 2/18  (11%)  Luminal B 23/65  (35%) | 26/114  (23%) | 2/12  (17%) | 22/78  (28%) | 15/176  (9%) |
| **HR+ / HER2+** | --- | --- | --- | 491/1636  (30%) | --- | 15/30  (25%) | 14/39  (36%) | 2/5  (40%) | 112/328  (34%) | 42/91  (46%) |
| **HR- / HER2+** | 29/59  (49%) | 134/207  (65%) | 83/131  (63%) | 516/1098  (47%) | 21/57  (37%) | 30/37  (81%) | 19/44  (43%) | 2/4  (50%) | 93/168  (55%) | 50/72  (69%) |
| **HR- / HER2-** | 24/26  (92%) | 84/170  (49%) | 54/106  (51%) | 829/2368 (35%) | 40/65  (62%) | 13/31  (42%) | 20/47  (43%) | 1/4  (25%) | 37/79  (47%) | 46/98  (47%) |

Supplementary 4 - Pathological complete response (breast and axilla) according to tumor subtypes (ypT0/ypTis/ypN0)

|  | **Zhang 2013** | **Boughey 2014** | **Tadros 2017** | **Fayanju 2018** | **Resende 2018** | **Cerbelli 2019** | **Kim 2019** | **Karalink 2021** | **Myers 2021** | **Jankowski 2023** |
| --- | --- | --- | --- | --- | --- | --- | --- | --- | --- | --- |
| **HR+ / HER2 -** | 17/145  (12%) | 36/317  (11%) | --- | 1134/8244  (14%) | 3/142  (2%) | Luminal A 0/18  (0%)  Luminal B 14/65  (21%) | 9/114  (8%) | 2/12  (17%) | 11/155  (7%) | 5/176  (3%) |
| **HR+ / HER2+** | --- | --- | --- | 1369/3628  (38%) | --- | 6/30  (20%) | 6/39  (15%) | 2/5  (40%) | 57/653  (9%) | 25/91  (27%) |
| **HR- / HER2+** | 44/101  (44%) | 94/207  (45%) | 89/263  (34%) | 1256/2147  (58%) | 19/74  (26%) | 22/37  (59%) | 13/44  (30%) | 2/4  (50%) | 49/343  (14%) | 36/72  (50%) |
| **HR- / HER2-** | 14/55  (25%) | 65/170  (38%) | 96/264  (36%) | 2611/6246  (30%) | 21/94  (22%) | 9/31  (29%) | 8/47  (17%) | 1/4  (25%) | 27/197  (14%) | 32/98  (33%) |
